# Cross-sectional study of *Fasciola gigantica* and other trematode infections of cattle in Edu Local Government Area, Kwara State, north-central Nigeria

**DOI:** 10.1186/s13071-016-1737-5

**Published:** 2016-08-26

**Authors:** Nusirat Elelu, Abdulganiyu Ambali, Gerald C. Coles, Mark C. Eisler

**Affiliations:** 1 University of Ilorin, Faculty of Veterinary Medicine, Ilorin, Kwara State Nigeria; 2 University of Bristol, School of Veterinary Science, Langford, Bristol BS40 5DU UK

**Keywords:** Trematodes, *Fasciola*, Paramphistomes, *Dicrocoelium*, *Schistosoma*, Nigeria, Kwara, Prevalence, Risk factors, FAMACHA©

## Abstract

**Background:**

Trematode infections of livestock are of global veterinary and public health importance causing serious economic losses. Majority of data on burden of trematode infections in Nigeria are based on abattoir surveys and there are very few data on herd level risk factors. The present study investigated the prevalence of, and herd level risk factors for, fasciolosis and other trematode infections in cattle in Edu Local Government Area (LGA).

**Methods:**

A cross-sectional survey used two-stage study design to investigate cattle belonging to 65 households. Two questionnaires were administered for household-level and individual cattle-level data. Faecal and blood samples were obtained from the cattle. Logistic regression analyses were performed to determine risk factors for infections.

**Results:**

Of 686 faecal samples analysed, 74.9 %, 16.1 %, 7.3 % and 1.2 % were positive for infections with *Fasciola gigantica,* paramphistomes, *Dicrocoelium hospes* and *Schistosoma bovis* respectively. *Fasciola gigantica* had higher prevalence in adult cattle (77.3 %) than weaners (62.5 %). Majority of co-infections was a combination of *F. gigantica* with paramphistomes 84/130 (64.6 %). Most (58.9 %) of the cattle belonged to FAMACHA© score 2. The mean packed cell volume (PCV) was 34.4 %. The sensitivity and specificity of FAMACHA© for anaemia (PCV < 24 %) were 18.2 and 96.9 %, respectively. Positive correlation was obtained between faecal egg counts for *F. gigantica* and paramphistomes (*R* = 0.15, *P* = 0.0001). Adult cattle were more likely to be infected with *F. gigantica* (odds ratio, OR: 1.94; Confidence Interval, CI: 1.19–3.16) than weaners. Cattle belonging to household heads aged between 40–59 years were more likely infected with paramphistomes (OR: 1.95; CI: 1.02–3.74) than those belonging to other age groups. Cattles from herds with size ≥ 100 were more likely infected with *D. hospes* than those from smaller herds (OR: 6.98; CI: 2.94–16.6).

**Conclusion:**

This study revealed high prevalence of infection with *F. gigantica* in Kwara State. The co-infections by *F. gigantica* and paramphistomes with a positive correlation should be considered during anthelmintic therapy. There is a need to optimise and validate the FAMACHA© for use in cattle based on breeds and variation in colour of ocular mucous membrane. Risk factors identified could assist in tailoring control strategies for various trematode infections to particular groups of farmers and cattle.

**Electronic supplementary material:**

The online version of this article (doi:10.1186/s13071-016-1737-5) contains supplementary material, which is available to authorized users.

## Background

Trematode infections cause serious economic losses to livestock globally. Many are zoonotic and thus a public health concern [1, 2]. Some of the trematode infections of cattle include species of *Fasciola*, *Dicrocoelium*, *Schistosoma* and paramphistomes. Fasciolosis due to *F. gigantica* has been reported in several parts of Africa [3–6] and Nigeria [7–10]. Other trematode infections in ruminants reported in Nigeria include species of *Dicrocoelium* [11], *Schistosoma* [12] and paramphistomes [7]. Trematode infections are known to cause clinical signs ranging from weight loss, sudden death [13] and anaemia in cattle [14, 15]. Tropical fasciolosis alone has been predicted to cause losses of about US$840 M per annum in the Africa’s 200 million cattle population [16] and this cost is likely to have increased significantly in the last sixteen years. Economic losses from fasciolosis may result directly from increased liver condemnation or indirectly from decreased livestock productivity [17]. Also about 165 million cattle are likely to be infected with *Schistosoma* spp. worldwide [18]. The cattle population in Nigeria is about 16 million [19] made up of predominantly humped zebu breeds (including the White Fulani, Sokoto Gudali and Red Bororo) and a limited number of hump less breeds including Keteku, Muturu and Kuri in the southwestern, southern and the northeastern parts, respectively [20]. They play a very important role in the Nigerian economy, contributing about 12.7 % of total agriculture gross domestic product (GDP) [21]. In the tropics, cattle are generally reared under the transhumance husbandry system with little supplementary feeding resulting in low productivity and high pre-weaning mortality [8]. Similarly, acute shortage of feeds during the dry season remains a common occurrence, compelling these animals to graze around water bodies that often contain large number of potential intermediate hosts of trematodes [8].

The majority of data on the burden of fasciolosis in Nigeria are based on abattoir surveys. However, there are very few data on the trematode prevalence in live cattle or on the herd level risk factors that may influence disease occurrence in Nigeria. Moreover, there are few recent data on infection of cattle with the other trematode species in Nigeria, and more recent information would be useful in formulating effective control strategies for this important group of parasites [7, 8, 22]. The present study investigated the prevalence of, and herd level risk factors for, fasciolosis and other trematode infections in cattle in the Edu Local Government Area (LGA).

## Methods

### Study location

**Fig. 1:**
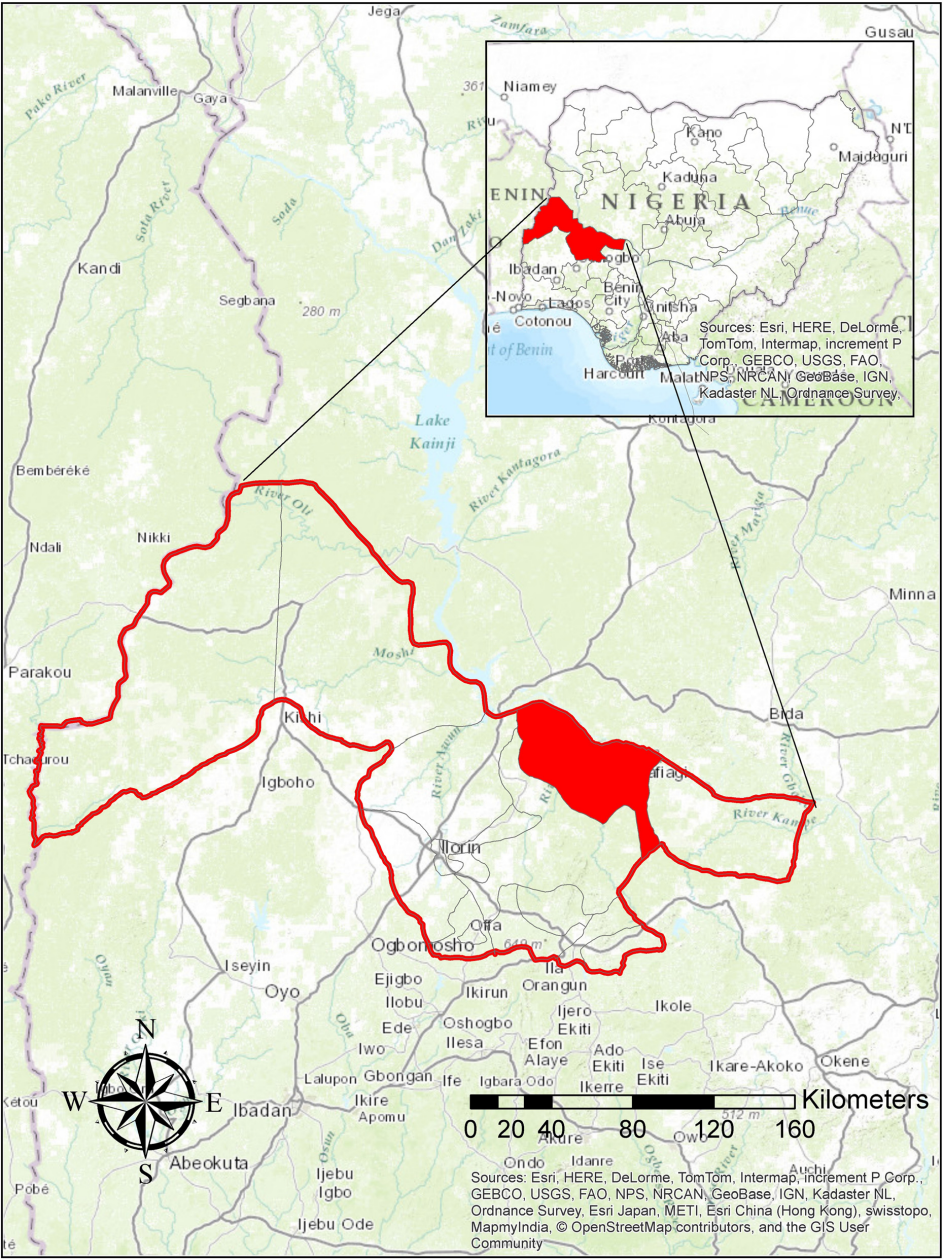
Map of Kwara State showing the location of Edu Local Government Area (study location). The inset map shows Kwara State within Nigeria

A cross-sectional study was conducted from May to August 2013 to determine the prevalence of trematode infections and herd level risk factors in cattle from 11 villages of Edu LGA, Kwara State, North-central Nigeria. Kwara State lies between 8°05′ and 10°15′N; and 2°73′ and 6°13′E (Fig. 1). It has a total area of about 34,500 square kilometres comprising rainforest in the south and wooded savannah in the larger part of the state. It has 16 local government areas. Rainfall has an annual range of 1,000–1,500 mm and average maximum temperature between 30 and 35 °C [23]. Edu LGA was selected as the study location because it has very large pastoralist settlements and is one of the largest area for cattle production in Kwara State. Rice, sugarcane and melon are the major crops planted. Because Edu LGA is bounded by the River Niger in the north, the area is often inundated with flood leading to devastating losses of livestock and farmland. The pastoralists therefore migrate uphill away from flood plains (starting in July) to neighbouring states once the rains begin. They do not return until the end of the year when the rains cease. A local informant identified 11 cattle producing villages in Edu LGA and these formed the sample population of this study.

### Study design and sampling

A two-stage sampling design was carried out. The first stage determined the number of households to be selected while the second stage determined the number of cattle to be sampled in each household. The number of households to be visited was calculated based on the formula: 1.96^2^ * P_exp_ (1-P_exp_)/ d^2^ [24] with an expected prevalence (P_exp_) of 50 % (no previous data on herd level prevalence in the area), 10 % desired precision and 95 % confidence interval (1.96). The second sampling stage to determine the number of cattle to be sampled per household and was applied to the two-stage sampling protocol of Cameron & Baldock [25] developed to determine presence of disease. The protocol was implemented by using the FreeCalc software version 2 [(c) Copyright 2001-Angus Cameron AusVet Animal Health Services]. The sensitivity (92.7 %) and specificity (94.9 %) of the Flukefinder ® [26], 40 % minimum expected prevalence and cattle population interval of 1–250 animals were included as parameters. A maximum of 13 animals are required to be sampled in each household. Only cattle ≥ 12 month-old were sampled to ensure they had experienced at least one complete grazing season.

A total of 686 cattle in 65 households were sampled for trematode infections. While the sample size calculations indicated that 96 households were required, there were only 65 accessible households present with cattle during the survey (others had either migrated uphill away from floodplains or were inaccessible due to the flood). All study locations were georeferenced using Garmin® global positioning system (GPS). Both faecal and blood samples were collected from the animals.

### Data collection

Two questionnaires were administered. The first focused on household level data such as farmer socio-demographic characteristics, management system, health practices, herd size, economic activities as well as knowledge and practice to control liver fluke disease. The second questionnaire was for individual cattle data and the information recorded included age, sex and breed. Both questionnaires were included in the analysis of herd level risk factors for trematode infections.

### Coprological analysis

Faecal samples were obtained directly from the rectum of each cow into sterile plastic gloves or from the ground if seen being produced. The glove were turned inside out, carefully tied, labelled and transported under cool conditions to the laboratory for analysis. The commercially available kit FlukeFinder® Richard Dixon ID, USA (http://www.flukefinder.com/) was used to isolate the trematode eggs by differential sieving and sedimentation according to manufacturer’s instructions.

The sensitivity and specificity of the Flukefinder® has been previously compared to other sedimentation methods [26]; this device has also been used previously to isolate trematode eggs [6, 27]. Two grams of individual cattle faecal sample were used and analysed according to the manufacturers’ instructions. The Flukefinder® is made up of two 2-in. wide sieves; these were washed thoroughly in between samples to prevent cross-contamination. The material was poured into a 50 mm Petri dish, three drops of methylene blue was added for contrast and then examined under a stereomicroscope at a magnification of 40×. Eggs were identified using standard keys [28]. Although the eggs of *F. gigantica* and paramphistomes are similar (both oval and operculated), the eggs of *F. gigantica* possess a distinct yellowish-brown colour and measure 156–197 μm in length and 90–104 μm in width compared with those of paramphistomes, which are clear in colour and measure 114–176 by 73–100 μm. Eggs of *D. hospes* are small (36–45 × 22–30 μm), oval, dark brown and operculate, with two characteristic dark “eye-spots” [28] and eggs of *S. bovis* are spindle-shaped with characteristic terminal spines on both sides of the non-operculate eggs [28].

### Haematological analysis

Blood samples for haematological analysis were collected from the jugular vein of the first three to four cattle sampled using EDTA anticoagulant (due to laboratory costs, blood samples were not obtained from all animals sampled in each household). The level of anaemia was also checked using the FAMACHA© anaemia chart to score the ocular mucous membrane [29]. The FAMCHA© chart is a low cost tool in determining anaemia status of ruminants [30]. The colour of ocular mucous membrane for each animal classified into five categories based on the FAMACHA© chart (ranging from bright red to pale) and recorded for individual animals. The packed cell volume (PCV) was also determined using the microhaematocrit method [31].

### Statistical analysis

Descriptive analyses were carried out on cattle data. Cattle that had at least one trematode egg count was considered positive. The overall prevalence (in %) at the animal level was the total number of cattle positive divided by the total number of cattle sampled in the study. Chi-square or Fisher’s exact tests were used to explore the relationships between the trematode prevalence, household and cattle data. Descriptive statistics for trematode co-infections are also presented.

The sensitivity and specificity of the FAMACHA© score compared to PCV were determined. Animals with FS scores of 1–3 were classified as non-anaemic while those with FS scores of 4–5 were grouped as anaemic. Reference value of 24–46 % for PCV in cattle was used [32]. The PCV values of ≤24 % were classified as anaemic and those above 25 % were considered non anaemic.

Pearson’s correlation coefficient was used to explore the relationships between trematode infections. In order to carry out correlation analyses, trematode egg counts were log-transformed [log(egg count +1)] to stabilise variances. The relationship between log-transformed trematode faecal egg counts and PCV was also determined.

Trematode infections and herd level risk factors were investigated by using predictor variables such as the household level factors, individual cattle data and haematological indices. The outcome variable was cattle trematode infection status expressed as a binary variable (0 = negative; 1 = positive). Variables with values for *P* < 0.20 from the Chi-square or Fisher’s exact test obtained from the coprological analysis were included in the binary logistic regression analysis. The risk factors were explored by binary logistic regression using the forward stepwise variable selection method and the 95 % confidence interval of odds ratio was calculated for the predictors [33].

Statistical analyses were carried out using Microsoft Excel® and IBM SPSS® statistics version 21.0 software (IBM Corp. 2012, Armonk, NY, USA). Distribution of trematode infections were also represented using the QGIS® spatial software version 2.2.

## Results

### Household and cattle data

A total of 65 households was surveyed (Additional file 1: Table S1). The respondents were all male with a mean age of 42.75 (SD ± 11.24, range 23–70) years. They were mostly married (96.9 %) and are of Fulani (83 %) ethnic origin. The majority had no formal education (93.5 %). They kept mainly livestock (67.7 %) comprising primarily cattle (90.5 %). The mean cattle herd size was 62.31 ± 46.26; cattle were kept mainly by pastoral/nomadic grazing system (78.5 %).

A total of 686 cattle was investigated using questionnaire surveys, comprising 318 (46.8 %) males and 362 (53.2 %) females. The mean age of cattle was 3.57 (SD ± 1.38) years with minimum and maximum ages of one and ten years, respectively. The breed composition was predominantly humped zebu 655 (95.9 %) with a very small minority made up of Jersey 27 (4.0 %) and a single Friesian (0.1 %).

### Coprological data

**Fig. 2:**
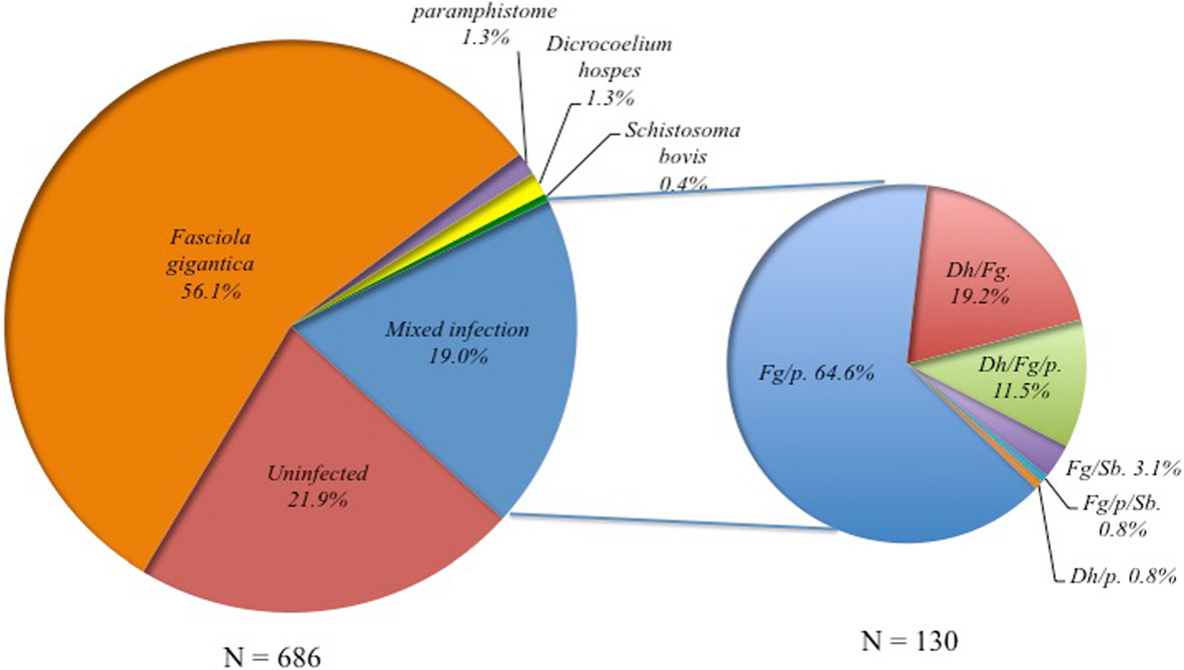
Proportions of single infections and co-infections by trematodes in cattle from the Edu Local Government Area of Kwara State, Nigeria

A total 686 faecal samples was analysed from cattle in 11 villages from which 536 (78.1 %) were positive for at least one of the parasites studied: 514 (74.9 %; 95 % CI: 72.0–79.0 %) for infections with *F. gigantica,* 110 (16.0 %; 95 % CI: 13.0–19.0 %) with paramphistomes, 50 (7.3 %; 95 % CI: 5.0–9.0 %) with *Dicrocoelium hospes* and 8 (1.2 %; 95 % CI: 0–2.0 %) with *Schistosoma bovi*s. Of these 686 cattle sampled, 406 (75.7 %) had single species trematode infections of which 385/686 (56.1 %) were *F. gigantica*, 9/686 (1.3 %) paramphistome, 9/686 (1.3 %) *D. hospes* and 3/686 (0.4 %) *S. bovis*. One hundred and thirty cattle 130/386 (19.0 %) had trematode co-infections and 150 cattle were uninfected (Fig. 2). Of the 130 cattle with co-infections, 84/130 (64.6 %), 25/130 (19.2 %) and 4 (3.1 %) of the animals had co-infection of *F. gigantica* with paramphistomes, *D. hospes* and *S. bovis*, respectively. Fifteen cattle had co-infection with three species of trematode: 15/130 (11.5 %) with *F. gigantica/* paramphistomes*/ D. hospes* and 1 (0.8 %) with *F. gigantica/* paramphistomes*/ S. bovis*, but none were infected with all four species. Finally, one animal had a co-infection with paramphistomes and *D. hospes* 1 (0.8 %) but not with *F. gigantica*.

**Fig. 3:**
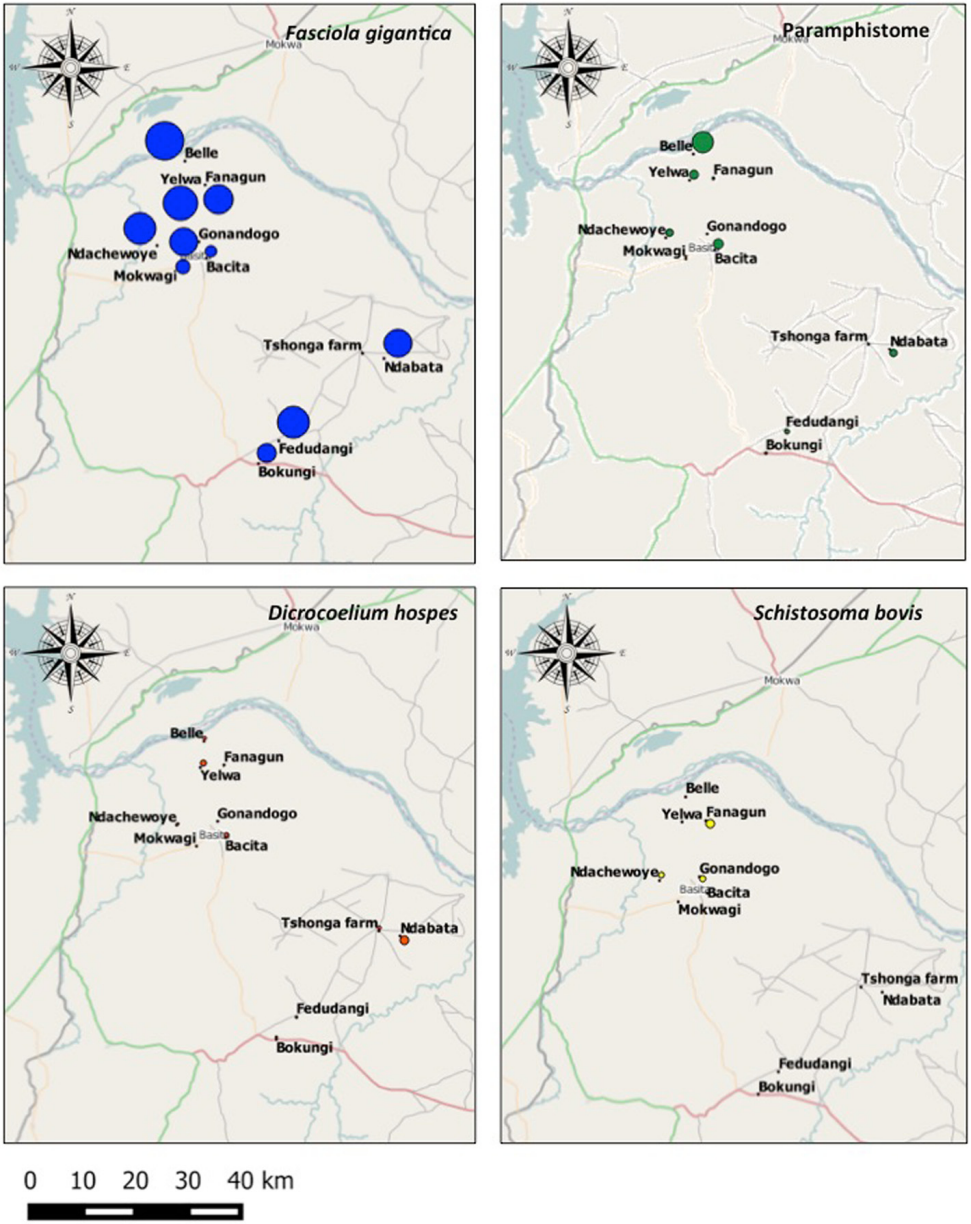
Distribution of trematode prevalence across villages sampled in Edu Local Government Area of Kwara State, North-central Nigeria. The size of the circles is proportional to prevalence: small circles (0 %); largest circle (100 %)

Infections with *F. gigantica* were found in cattle in all villages at prevalences ranging from 3.6 to 100 %, with seven out of the eleven villages having prevalence greater than 70 % (Table 1). The highest prevalence was recorded in Belle (100 %), Yelwa (91.3 %), Fedudangi (85.7 %) and Ndachewoye (85.2 %) whereas the lowest prevalence was recorded in Tshonga farm (3.6 %). Paramphistome infections were also found in all eleven villages, and *D. hospes* infections in all but one village (Mokwagi). The highest prevalences for paramphistomes and *D. hospes* were 58.3 % (Belle village) and 24.0 % (Ndabata), respectively. Three out of the eleven villages studied (Fanagun, Gonandogo and Ndachewoye) were positive for infection with *S. bovis* with prevalence rates of less than 2 %. Distribution maps of the prevalences of the various trematode species are shown in Fig. 3.

**Table 1:** Households and cattle trematode infections from villages studied in Edu LGA, Kwara State, Nigeria

		Households					Cattle								
Village	Altitude (m)	*F. gigantica*		Paramphistomes	*D. hospes*	*S. bovis*		*F. gigantica*		Paramphistomes		*D. hospes*		*S. bovis*	
		*N*	*n*	*N*	*N*	*N*	*N*	*n*	(%)	*n*	(%)	*n*	(%)	*n*	(%)
Bacita	106	2	2	2	2	0	22	7	(31.8)	6	(27.3)	3	(13.6)	0	(0.0)
Belle	76	5	5	5	1	0	48	48	(100)	28	(58.3)	6	(12.5)	0	(0.0)
Bokungi	221	3	3	3	2	0	23	12	(52.2)	2	(8.7)	2	(8.7)	0	(0.0)
Fanagun	82	14	14	7	5	3	158	122	(77.2)	12	(7.6)	9	(5.7)	3	(1.9)
Fedudangi	193	3	3	1	0	0	14	12	(85.7)	2	(14.3)	1	(7.1)	0	(0.0)
Gonandogo	84	10	10	4	4	2	136	100	(73.5)	2	(1.5)	3	(2.2)	2	(1.5)
Mokwagi	114	1	1	1	0	0	13	5	(38.5)	1	(7.7)	0	(0.0)	0	(0.0)
Ndabata	202	2	2	1	2	0	25	19	(76.0)	5	(20.0)	6	(24.0)	0	(0.0)
Ndachewoye	98	21	21	17	9	2	196	167	(85.2)	45	(23.0)	13	(6.6)	3	(1.5)
Tshonga Farm	175	2	1	1	2	0	28	1	(3.6)	1	(3.6)	3	(10.7)	0	(0.0)
Yelwa	77	2	2	2	2	0	23	21	(91.3)	6	(26.1)	4	(17.4)	0	(0.0)
Total		65	64	44	29	7	686	514	(74.9)	110	(16.0)	50	(7.3)	8	(1.2)

*Abbreviations*: N number of samples, *n* number of infected, *(%)* prevalence

Faecal egg counts ranged from 0–73 (mean 5.92) eggs per gram (epg) for *F. gigantica*, 0–10 (mean 0.44) epg for paramphistomes, 0–7 (mean 0.15) epg for *D. hospes* and 0–4 epg for *S. bovis. Fasciola gigantica* had higher prevalence in adult cattle (77.3 %) than in those younger than two years (62.5 %) (*χ*^2^ = 0.002, *P* < 0.05). The differences in prevalence between age groups were statistically significant for *F. gigantica* (*χ*^2^ = 0.002, *P* < 0.05). The results of the univariate analyses are presented in Additional file 2: Table S2). The prevalence of *S. bovis* infection was too low to carry out further meaningful statistical analyses.

### Correlations between trematode infections of cattle

A significant positive correlation was obtained between log-transformed data for faecal egg counts for *F. gigantica* and paramphistomes (*R*^2^ = 0.023, *P* = 0.0001) but there were no significant correlations between faecal egg counts for *F. gigantica* and *D. hospes* (*R*^2^ = 0.00003, *P* = 0.906) or between faecal egg counts for paramphistomes and *D. hospes* (*R*^2^ = 0.004, *P* = 0.088).

### Haematological data

A total of 217 whole blood samples were analysed. The packed cell volume (PCV) varied within a range of 12–48 % with a mean value of 34.9 % (SD ± 7.25 %; normal range in cattle 24–46 %) [15]. Twenty-two (10.1 %) of these cattle had PCVs less than the lower normal limit of 24 %. The frequency distribution (Table 2) of FAMACHA® score revealed that the majority 58.9 % (128/217) of the cattle studied belonged to FS2 with the least being FS4. None of the cattle studied had a FS score 5.

**Table 2:** Mean packed cell volume (PCV) of cattle in different categories of FAMACHA© score

FAMACHA© score	Number of cattle	Mean PCV	Range PCV
	*N* (% of total)		
1	56 (25.8)	35.9	22–48
2	128 (58.9)	34.9	20–48
3	23 (24.4)	30.5	20–46
4	10 (4.6)	27.7	12–41

Total number of cattle: 217

The sensitivity and specificity of the FAMACHA© ocular score in cattle were 18.2 % (4/22) and 96.9 % (189/195), respectively using PCV ≤ 24 % and FS of 4–5 as cut-off values for anaemia (Table 3).

**Table 3:** Two-by-two contingency table of PCV and FAMACHA© score of cattle

		FAMACHA© Score		
		FS 4–5 (Anaemic)	FS 1–3 (Non-anaemic)	Total
PCV	≤ 24 % (Anaemic)	4	18	22
	> 24 % (Non-anaemic)	6	189	195
	Total	10	207	217

Sensitivity: 18.2 %; Specificity: 96.9 %

### Correlation between trematode infections and PCV in cattle

The correlation analysis between log-transformed trematode faecal egg counts and PCV revealed a weak negative but statistically non-significant correlation between PCV and *F. gigantica* (*R* = -0.050, *R*^2^ = 0.0023, *P* = 0.463) as well as between PCV and *D. hospes* (*R* = -0.070, *R*^*2*^ = 0.0049, *P* = 0.305).

### Herd level risk factors of trematode infections

Cattle herd sizes greater than 100 were less likely to have *F. gigantica* infections than those of smaller herds (odds ratio OR: 0.28; 95 % confidence interval CI: 0.14–0.58). Adult cattle (≥ 2 years), were more likely to be infected (OR: 1.94; CI: 1.19–3.16) than younger cattle. Cattle belonging to heads of households aged between 40–59 years were more likely to be infected with paramphistomes (OR: 1.95; CI: 1.02–3.74) than respondents of other age groups (20–39 and > 60 years), while those belonging to the ‘Zabaruma’ ethnic group were less likely infected (OR: 0.05; CI: 0.01–0.22) than the Fulani or Zimbabwean farmers. Cattle herd size greater than 100 were more likely to be infected with *D. hospes* than those in smaller herds (OR: 6.98; CI: 2.94–16.6). Logistic regression could not be performed on *S. bovis* infection because there were too few infected animals (Table 4).

**Table 4:** Binary logistic regression to investigate risk factors for cattle trematode infections in Kwara State, Nigeria

Parasite	Risk factor	Odds ratio	95 % CI	*P*
*F. gigantica*	Cattle herd size > 100	0.28	0.14–0.58	0.001
	Adult cattle (≥ 2 years)	1.94	1.19–3.16	0.008
Paramphistomes	Ethnicity of head of respondents	0.05	0.01–0.22	0.001
	Head of respondents (40–59 years)	1.95	1.02–3.74	0.043
*D. hospes*	Cattle herd size > 100	6.98	2.94–16.6	0.001

## Discussion

This is the first reported study to determine the burden of trematode infections in live cattle in Kwara state, North-central, Nigeria. Zebu cattle were the most predominant breeds because they are the predominant breed in Nigeria kept primarily for milk and beef [34]. The few exotic breeds sampled were from the government owned intensive farm in Tshonga district. There were more female (53.5 %) animals sampled than males (46.5 %) because more female animals, are usually kept by farmers for herd growth and milk production [35].

On the basis of coprological examination, trematode infections were common in cattle in Edu LGA, occurring in 78.1 % of animals investigated. The most frequent species of trematode identified was *Fasciola gigantica* in 74.9 % of cattle. This is considerably higher than the 22.5 % prevalence reported in an abattoir study in the state capital [36] but similar to a study carried out in Bauchi State of northern Nigeria reporting a prevalence of 76.9 % [37].

Egg counts for *F. gigantica* observed in our study (range 0–73 epg) were considerably lower than reported in previous studies in zebu cattle, for example, a range of 400–1,100 epg in faeces of cattle in Bangladesh [38], a mean of 81.2 egg per 2 g of faeces in Ethiopia [39] and range of 0*–*167 epg in Tanzania [6]. The values from the present study were however higher than those reported in Ghana [40] and in a previous abattoir study in Nigeria [41]. Further experimental studies are recommended to determine the egg output index of adult *F. gigantica* in Nigeria and Africa. Fasciolosis causes losses in livestock due to losses from mortality and reduced productivity and is one of the leading causes of liver condemnation in abattoir in Kwara state [36].

Fasciolosis is also an important zoonotic disease [42]. Ndachewoye houses a large abandoned dam that was used by the Bacita sugar factory. It serves as a watering point for both cattle and humans. It is an all year round dam that could favour the life cycle of *F. gigantica* and other trematode species. Transmission studies on *Fasciola* spp. revealed that some free-floating metacercariae might be suspended in water where they can be ingested by the definitive hosts [43]. Moreover, human fasciolosis has been reported to occur from eating uncooked watercress derived from endemic areas where infected cattle range freely and probably from contaminated water [44, 45].

The other trematodes prevalent in this study were *D. hospes*, *S. bovis* and paramphistomes. The prevalence of paramphistomes recorded here were lower than in previous reports from southern Ghana [40], Zambia [46], Tanzania [6] but higher than reported in Turkey [47]. Paramphistome species has been reported in Nigeria with up to 2,000 adult parasite in cattle [48].

Only a few studies are available on *D. hospes* infection in Nigeria the majority representing abattoir surveys [7, 8]. Although the clinical disease when present can lead to severe anaemia, oedema and emaciation, it is usually asymptomatic [49]. A prepatent period of up to 59 days post experimental infection with *Dicrocoelium dendriticum* has been reported in lambs, hence the low faecal egg count (range 0–7 epg) reported in this study may not fully reflect the level of infection [50]. The prevalence (7.3 %) reported in this study is considerably lower than the 38 % previously reported in cattle at slaughter in Zaria, Nigeria [8].

Infections with *S. bovis* was reported in only three of the villages studied (Gonandogo, Ndachewoye and Fanagun) with low prevalences (< 2 %) similar to those observed in a recent study in Tanzania [6]. However, another study in Zambia, reported up to 22 % prevalence for bovine schistosomiasis [51].

There was a significantly higher prevalence of *F. gigantica* infections in adult cattle (77.3 %) than weaners (62.5 %). This is in agreement with a study in Tanzania with similar findings [6] and may be due to longer exposure of adult animals to infection [52].

Although some past studies revealed higher trematode prevalence rates in female than in male animals [40, 53], no significant difference was found in this study. This finding agrees with a previous study in Uganda that reported no significant difference in *F. gigantica* prevalence between sexes [27].

Out of the 686 faecal samples analysed, 130 (19.0 %) were from cattle with trematode co-infections, the majority of which were *F. gigantica* co-infecting with paramphistomes (64.6 %). This may reflect the similarity of the life-cycles of these parasites, which require lymnaeid snails as intermediate hosts [54, 55]. The metacercariae of both trematodes could be found in the same places and might be ingested together by the cattle [56]. This finding was also supported by a significant positive correlation between *F. gigantica* and paramphistomes faecal egg counts. This positive relationship between the two parasites has been reported previously [46, 51, 57].

Although the haematological results obtained from this study indicated the mean PCV of 34.5 % was within normal range for cattle, which is 24–46 % [32], anaemia due to fasciolosis has been attributed to blood-sucking by adult parasites over long period of time in chronic infections [38, 58]. Failure to demonstrate any effect of trematode infection on PCV may have been due to improved nutritional status of the cattle during the rainy season when they were sampled. The nutritional status of cattle has been previously reported to influence the effect of liver fluke disease [59].

The FAMCHA© chart is a low cost tool for determining anaemia status in ruminants [60, 61] and can be used as part of an integrated worm control program [61]. Selective treatment of animals based on anaemic status is important in preventing anthelmintic resistance [29]. The usefulness of the FAMACHA© chart been evaluated previously in small ruminants in Nigeria [62, 63]. Using scores of 4 or 5 as being indicative of anaemia, the FAMACHA© chart gave a low sensitivity (18.2 %) compared with PCV of ≤ 24 % as a gold standard indicator for anaemia. The PCV cut-off value used to represent anaemia has a significant impact on sensitivity and specificity; values of 64.1 % and 91.3 %, respectively, have been reported in sheep using FAMACHA© score 4 or 5 and PCV ≤ 19 % [30], but there were too few cattle in our study with low enough PCVs for comparable analysis. The FAMACHA© was designed specifically for use in sheep with haemonchosis, optimising the chart for use in cattle based on breeds and variation in colour of ocular mucous membrane might be rewarding.

Analysis of risk factors for trematode infections indicated that cattle in large herds were significantly less likely to be infected with *F. gigantica* than those from smaller herds. This is because of the possibility of better herd management such as routine anthelmintic treatment. The risk of *F. gigantica* infection has been previously shown to be lower in medium and large sized non-dairy Danish cattle herds managed intensively [64]. This was however not the case with *D. hospes* infection where large cattle herds were seven times more likely to be infected than small herds. Other factors such as improper dosing of drugs, anthelmintic resistance or other management system factors may therefore predispose to *D. hospes* infection. The age and ethnicity of respondents proved to be important in predicting paramphistome infection, cattle belonging to respondents aged 40–59 years of age were 1.95 times more likely to have paramphistomes than cattle belonging to respondents of other age groups, and cattle of responents of Zabaruma ethnic groups were less likely to have paramphistomosis. These ethnic groups keep their cattle semi-intensively, and therefore are less likely to come in contact with infected pasture.

## Conclusions

This study revealed the presence of infections with *F. gigantica*, *D. hospes*, *S. bovis* and paramphistomes in cattle sampled from Edu, Kwara State. There was a high variability in the prevalence of trematodes across villages and this may be important in successful control of the parasites. The high prevalence recorded for *F. gigantica* suggests that an anthelmintic resistance survey on the currently available drugs in the state would be advisable. The high positive correlation between *F. gigantica* and paramphistome infections and hence likelihood of co-infections should be considered when carrying out anthelmintic therapy. The drugs of choice should be effective against both parasites. There is also a need to optimise and validate the FAMACHA© chart for use in cattle based on breeds and variation in colour of ocular mucous membrane. This would improve its usefulness in identifying cattle with anaemia as a means of selective treatment and hence reduce anthelmintic use/resistance. Finally the risk factors identified in this survey such as herd size and cattle age could assist in tailoring control strategies for various trematode infections to particular group of farmers and cattle.

## Abbreviations

EDTA, Ethylene diamine tetra acetic acid; epg, eggs per gram; FS, FAMACHA© score; GDP, Gross Domestic Product; GPS, Global positioning system; LGA, Local Government Area; PCV, Packed cell volume; QGIS, Quantum geographic information system; SD, Standard deviation; Sp, Specificity; Ss, Sensitivity; UIN, University investigation number

## Additional files

Additional file 1: Table S1. General characterisitics of households surveyed in Edu LGA, Kwara State. (XLSX 10 kb) Additional file 2: Table S2. Univariate analysis of trematode infections in cattle based on household and cattle data in Edu, Kwara State, Nigeria. (XLSX 16 kb)
